# Multicolor spectral photon-counting computed tomography: *in vivo* dual contrast imaging with a high count rate scanner

**DOI:** 10.1038/s41598-017-04659-9

**Published:** 2017-07-06

**Authors:** David P. Cormode, Salim Si-Mohamed, Daniel Bar-Ness, Monica Sigovan, Pratap C. Naha, Joelle Balegamire, Franck Lavenne, Philippe Coulon, Ewald Roessl, Matthias Bartels, Michal Rokni, Ira Blevis, Loic Boussel, Philippe Douek

**Affiliations:** 1 Department of Radiology, University of Pennsylvania, Philadelphia, PA USA; 2 Radiology Department, Hospices Civils de Lyon, Lyon, France; 3 CREATIS, UMR CNRS 5220, Inserm U1044, University Lyon1 Claude Bernard, Lyon, France; 4 LAGEP Laboratory, University Lyon 1 Claude Bernard, Lyon, France; 5 CERMEP, Lyon, France; 6 CT Clinical Science, Philips, Suresnes France; 7 Philips GmbH Innovative Technologies, Research Laboratories, Hamburg, Germany; 8 Global Advanced Technologies, CT, Philips, Haifa Israel

## Abstract

A new prototype spectral photon-counting computed tomography (SPCCT) based on a modified clinical CT system has been developed. SPCCT analysis of the energy composition of the transmitted x-ray spectrum potentially allows simultaneous dual contrast agent imaging, however, this has not yet been demonstrated with such a system. We investigated the feasibility of using this system to distinguish gold nanoparticles (AuNP) and an iodinated contrast agent. The contrast agents and calcium phosphate were imaged in phantoms. Conventional CT, gold K-edge, iodine and water images were produced and demonstrated accurate discrimination and quantification of gold and iodine concentrations in a phantom containing mixtures of the contrast agents. *In vivo* experiments were performed using New Zealand White rabbits at several times points after injections of AuNP and iodinated contrast agents. We found that the contrast material maps clearly differentiated the distributions of gold and iodine in the tissues allowing quantification of the contrast agents’ concentrations, which matched their expected pharmacokinetics. Furthermore, rapid, repetitive scanning was done, which allowed measurement of contrast agent kinetics with high temporal resolution. In conclusion, a clinical scale, high count rate SPCCT system is able to discriminate gold and iodine contrast media in different organs *in vivo*.

## Introduction

**Figure 1:**
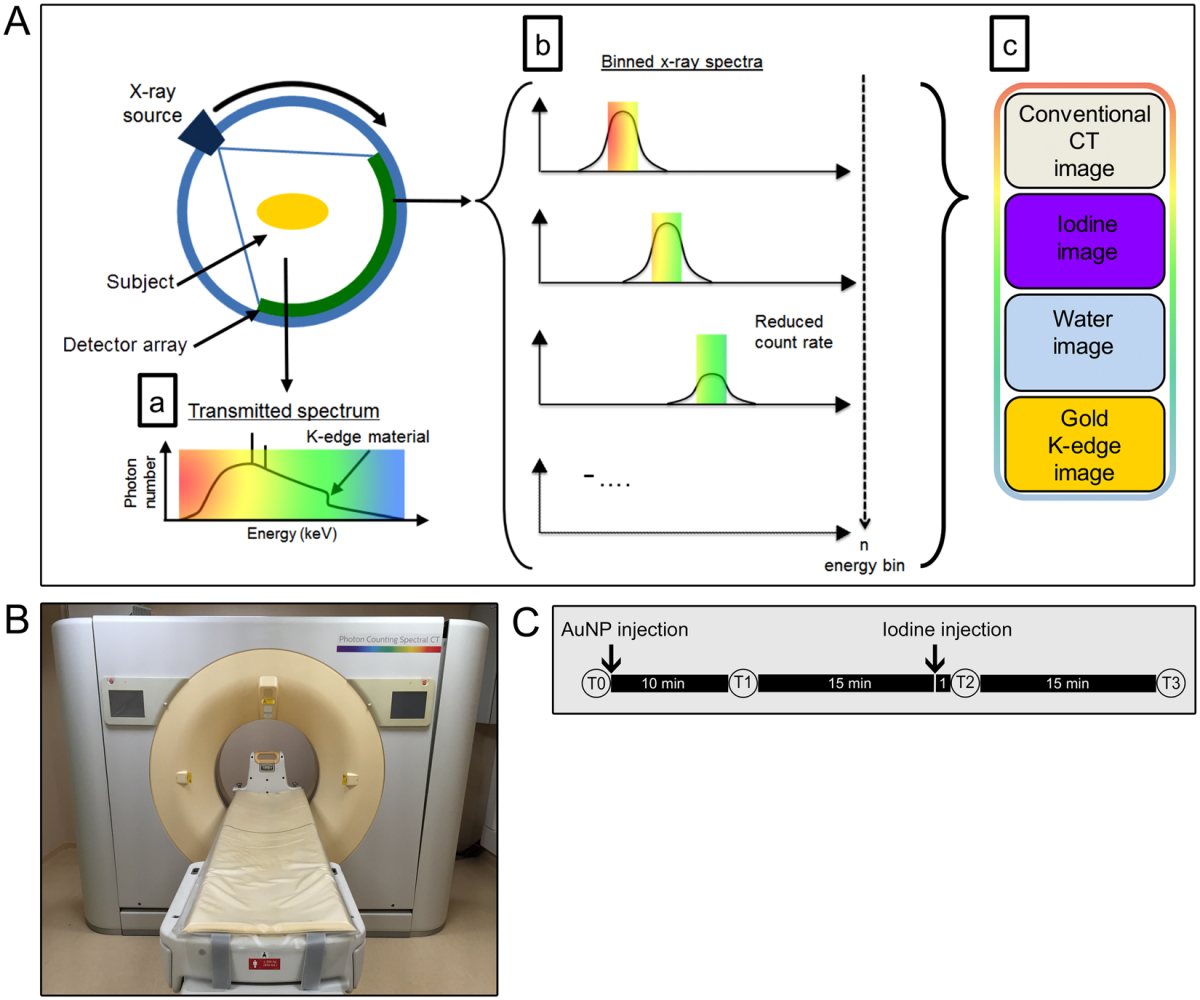
(**A**) Schematic depiction of SPCCT image formation. The transmitted spectrum (a) is divided by photon-counting detectors into multiple bins (b). These datasets are jointly processed to provide conventional images, and specific material decomposition images, e.g. water, iodine and gold images (c). (**B**) Photograph of the spectral photon-counting CT system used in this study. (**C**) Schematic depiction of the *in vivo* imaging protocol.

Spectral photon-counting computed tomography (SPCCT) is a new imaging modality that is currently heavily investigated. SPCCT scanners use a standard polychromatic x-ray source and dedicated photon-counting detectors that discriminate the transmitted photons based on their energy and separate them into several energy bins^1–7^. Placing the energy bin boundaries in close proximity to the K-edge energies of elements allows specific imaging of these elements, which is also known as K-edge imaging (Fig. 1A). This technology can be used to distinguish several different materials in the field of view simultaneously, referred to as multicolor CT imaging^8–10^. The contrast agents reported for this technique have been based on heavy atoms, due to their K-edge energies being within a region of high x-ray flux within the beams used (50–90 keV), such as gold, bismuth, gadolinium and ytterbium^10–17^. Moreover, the spectral information can be additionally used for material separation in a similar fashion to dual-energy CT^3, 18^, i.e. allowing the formation of water or iodine density maps.

Spectral photon-counting CT scanners have been used to facilitate CT-based molecular imaging, as only a single scan, performed post-injection, is needed to detect accumulations of contrast agent in the target site, eliminating the need for comparison of pre- and post-injection images^8, 13, 17^. For example, SPCCT has been used to image macrophages inside atherosclerotic plaque with the use of gold nanoparticles, and simultaneously image the arterial lumen with an iodine contrast agent^19^. However, these previous results were obtained using SPCCT systems designed for small animal imaging and that had several technological limitations incompatible with clinical imaging, such as low temporal resolution, very long acquisition time due to low count rate performance and limited detector collimation^20^.

Recently, prototype clinical scale SPCCT scanners have been built^21^. Some of the characteristics of these scanners have been studied and initial patient imaging results have been reported^21–23^. However, it is not known if these clinical scale scanners retain the valuable ability to distinguish multiple materials simultaneously (e.g. iodine, gold and tissue). The purpose of this study was to investigate whether a small FOV prototype SPCCT scanner based on a modified clinical CT system (Fig. 1B) with high count rate performance was able to distinguish iodine and gold contrast media from endogenous tissues in a single scan.

## Results

### AuNP characterization

**Figure 2:**
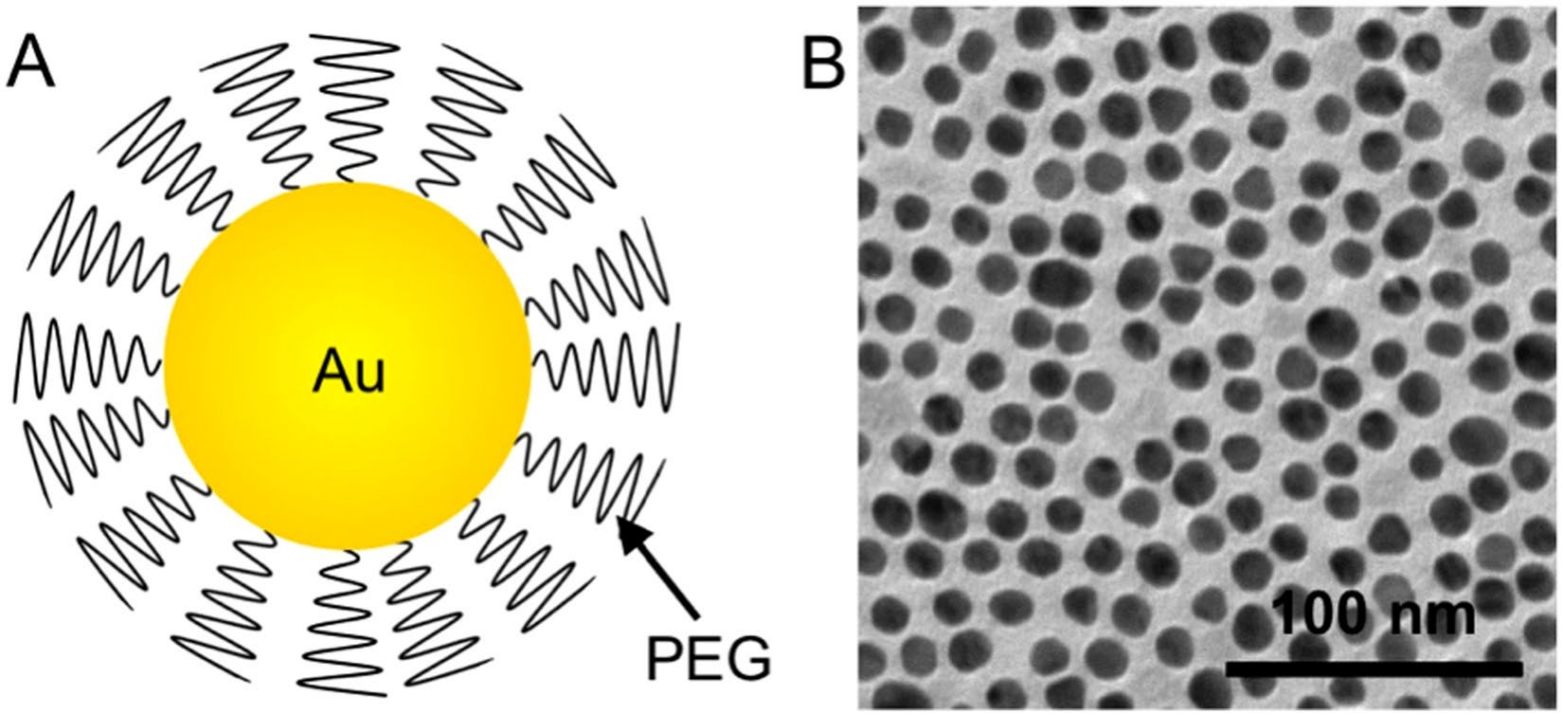
(**A**) Schematic representation of the gold nanoparticle used in this report. (**B**) TEM of the gold nanoparticles.

The average core diameter of the gold nanoparticles (AuNP) was found from transmission electron microscopy to be 12.5 +/− 2.4 nm (Fig. 2), while their mean hydrodynamic diameter was 18.3 +/− 0.5 nm, as determined by dynamic light scattering.

### Phantom imaging

**Figure 3:**
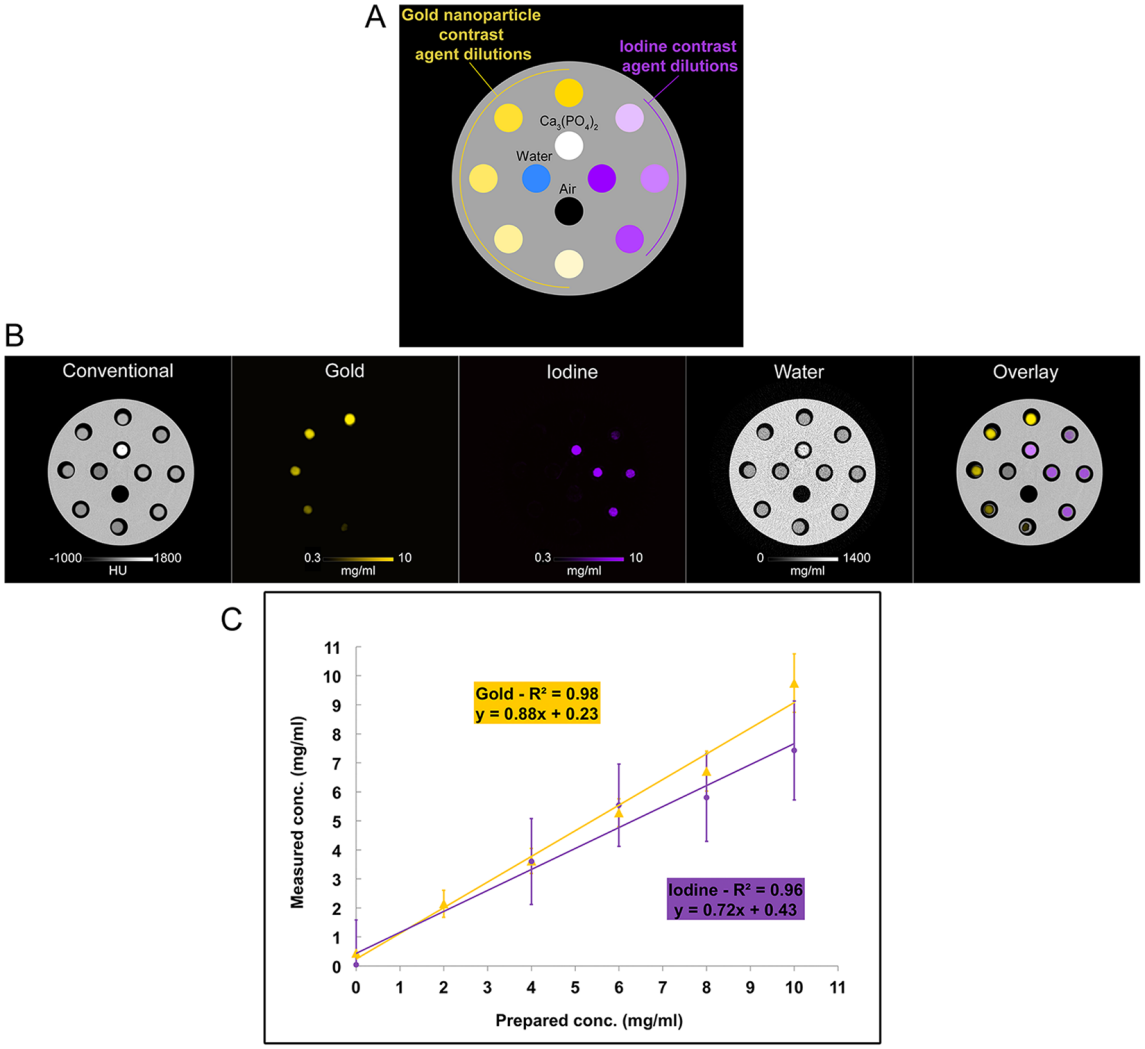
(**A**) Schematic of the phantom with unmixed solutions. (**B**) Conventional, gold, iodine, water and overlay images are depicted. (**C**) Graph of the expected and the measured concentrations.

In order to study the material discriminating capabilities of this SPCCT scanner, we first imaged a phantom contained a range of concentrations of AuNP and iodine (Fig. 3). As can be seen, the scanner could accurately determine the location of gold and iodine in the field of view (Fig. 3A,B). Since the plastic of the phantom is composed of elements close in atomic weight to those that make up water, both the plastic of the phantom and water in the vials appear in the water image. Discrimination between iodine and calcium was not attempted in this study. We therefore observed signal arising from the calcium phosphate sample in both the iodine and water images (Fig. 3B). There was a linear correlation between the contrast agent concentration and the signal produced in the element specific images (Fig. 3C). However, the concentration determined from the images was typically slightly underestimated compared to the actual concentration (for gold the slope of the line was 0.9, while for iodine it was 0.7). Bland-Altman plots for this phantom are shown in Supporting Fig. 1.

**Figure 4:**
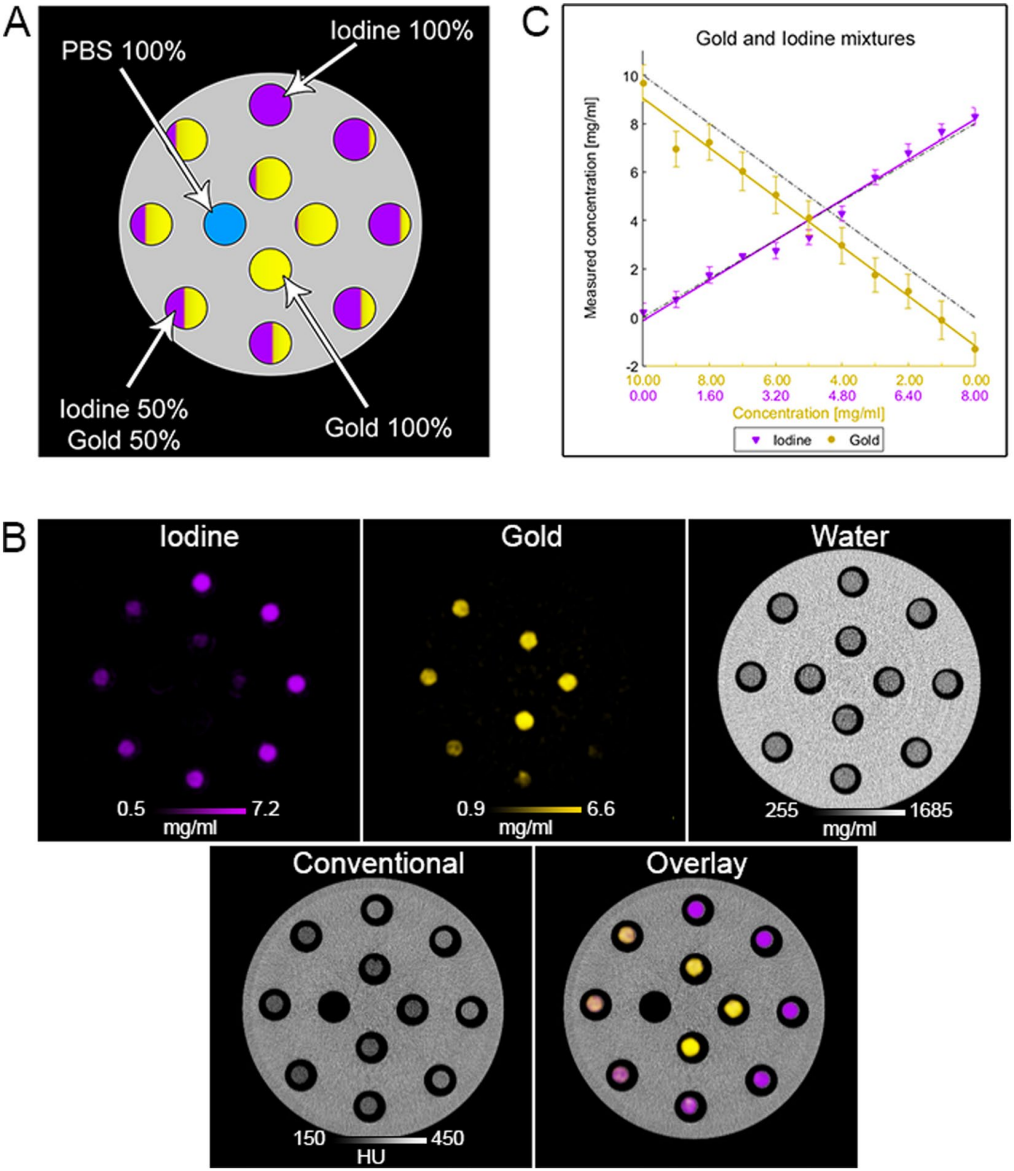
(**A**) Schematic of the phantom with mixed solutions. (**B**) Conventional, gold, iodine, water and overlay images are depicted. (**C**) Comparison of the expected and the measured concentrations.

In the second phantom, as expected, different solutions of mixed contrast agents could not be differentiated on conventional CT images as all samples had similar attenuations with an average of 279 ± 10 HU (Fig. 4). However, we found that the contrast agents were accurately identified in the gold and iodine specific images (Fig. 4B). As above, there was a linear correlation between the SPCCT measurements and the known concentrations of iodine and gold (Fig. 4C). However, the analysis for gold revealed an offset of −1.16 mg/ml. The phantom data indicates a detection limit close to 1 mg/ml of contrast agent, similar to conventional CT systems^24^, but with the added benefit of material discrimination. Note that the size of the sample is typically slightly larger in the water image than in the gold or iodine images since the plastic rim of the tube appears in the water image, but not in the material specific images. Bland-Altman plots for this phantom are shown in Supporting Fig. 2. The noise in the iodine image, for example, was 0.35 mg/ml, resulting in CNR of 24.9 in the sample with the highest concentration. Similar results were found for the gold images.

### *In vivo* imaging

**Figure 5:**
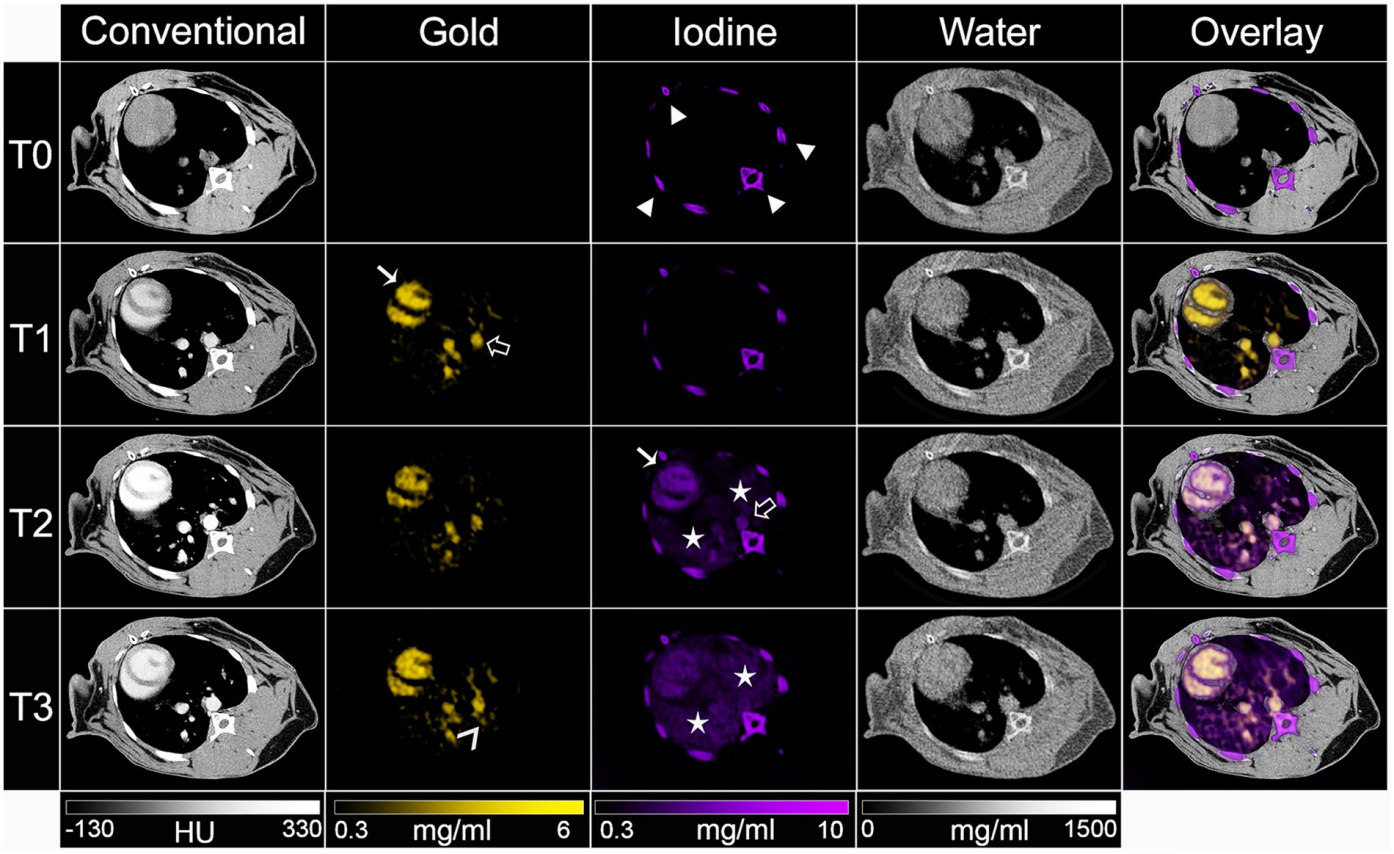
Photon-counting CT images of the chest of a rabbit. Conventional, gold, iodine, water and overlay images are shown for the three different time points. Arrowheads indicate bones, white arrows indicate the heart, black arrows indicate the aorta and stars indicate the lungs.

**Figure 6:**
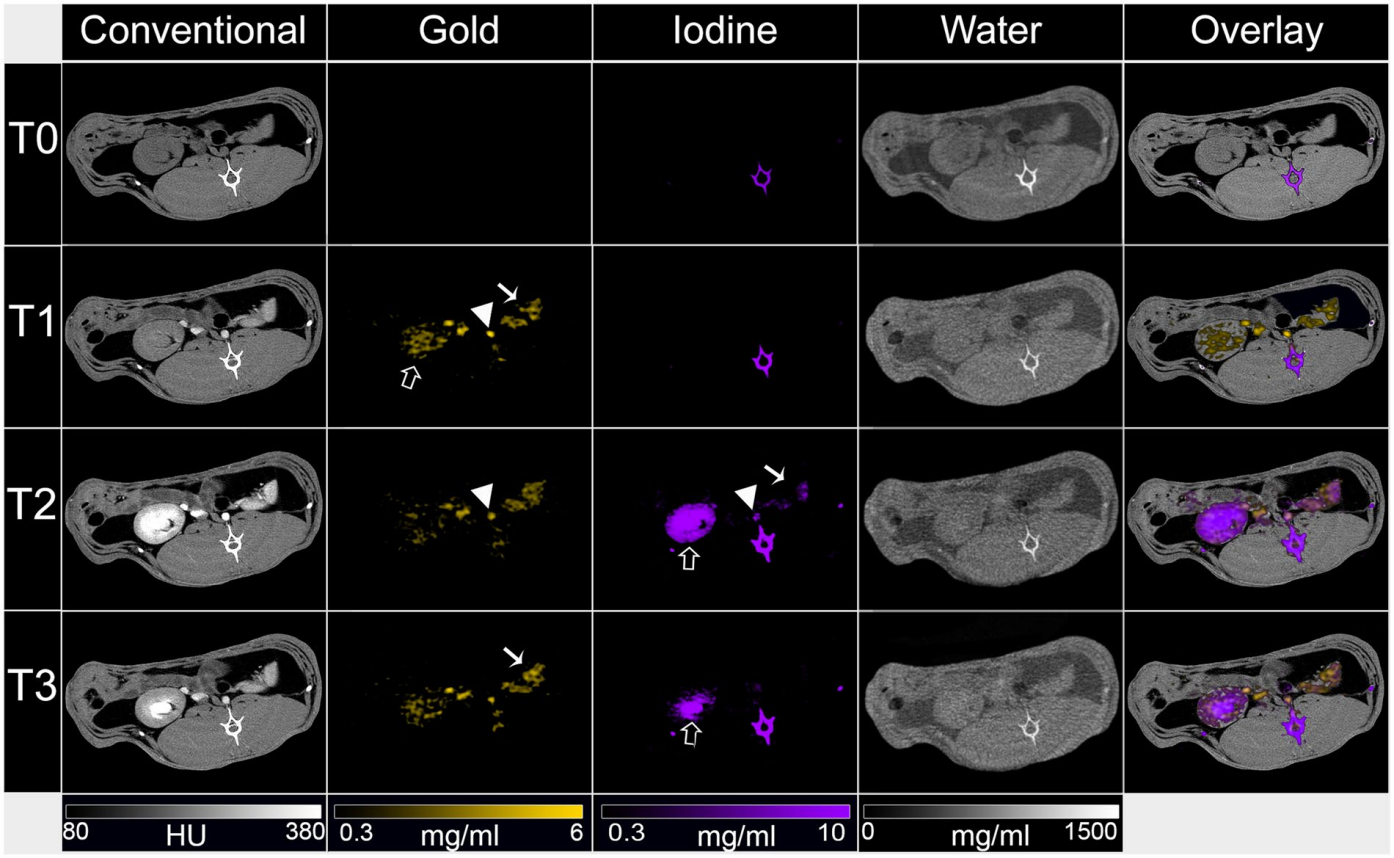
Photon-counting CT images of the abdomen of a rabbit. Conventional, gold, iodine, water and overlay images are shown for the three different time points. Arrowheads indicate aorta, white arrows indicate the spleen and black arrows indicate the kidney.

*In vivo* testing of the material discrimination capabilities of the scanner was done via injecting rabbits with both AuNP and subsequently an iodinated contrast agent (see timeline in Fig. 1C). The agents used were selected such as they had differing pharmacokinetics and biodistributions (iodinated contrast media are swiftly excreted via the urine, while the AuNP used are a blood pool agent with a long blood residence time^25^). The difference in pharmacokinetics helped confirm the imaging results, as will be seen later. *In vivo* spectral CT images (conventional CT, gold, iodine, water and overlay) are shown in Figs 5 and 6.

As can be seen from the images and from the image analysis, variations in attenuation were seen in the blood, kidney, spleen and renal pelvis at the different time points. For example, the attenuation in the blood increased from T0 to T1 after AuNP injection and increased further at T2, after iodine injection, before declining at T3. However, conventional CT images do not allow contrast arising from gold to be discriminated from that due to iodine. For example, at T3 in the abdomen (Fig. 6), contrast can be seen in the kidney and spleen, but it cannot be ascribed to one or the other or both of the contrast agents used.

**Figure 7:**
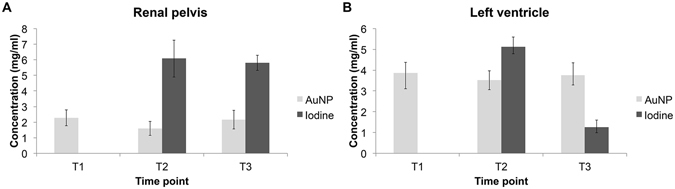
Concentrations of contrast agents in (**A**) the blood compartment and (**B**) the renal pelvis.

Gold images allowed discrimination of gold rich matter from all other tissues and materials. Strong signals were observed in the blood vessels, which were sustained over time (Figs 5 and 7). The signal of gold was also persistent in the liver, spleen and the kidney (Table 1), which is likely due in part to the presence of blood in these organs, over this timeframe. A low signal was seen in the renal pelvis.

**Table 1:** Values of attenuation and concentrations of gold and iodine in various organs.

Organ	Spectral Reconstruction	T1		T2		T3	
		Mean	SD	Mean	SD	Mean	SD
Left ventricle	Conventional (HU)	139.93	21.89	310.07	30.91	167.52	19.34
	Gold (mg/ml)	3.86	0.75	3.53	0.47	3.75	0.47
	Iodine (mg/ml)	★	★	5.12	0.33	1.26	0.27
Liver	Conventional (HU)	77.17	23.90	118.30	30.34	92.31	29.27
	Gold (mg/ml)	1.80	0.92	1.31	0.50	1.28	0.94
	Iodine (mg/ml)	★	★	1.69	0.65	0.45	0.61
Spleen	Conventional (HU)	75.36	25.57	164.33	43.27	98.88	25.52
	Gold (mg/ml)	2.01	0.80	1.66	0.63	1.72	0.70
	Iodine (mg/ml)	★	★	2.80	0.72	0.88	0.50
Kidney	Conventional (HU)	63.77	24.81	139.17	28.84	109.98	24.40
	Gold (mg/ml)	1.49	1.63	1.41	0.67	1.53	0.63
	Iodine (mg/ml)	★	★	2.20	0.69	1.35	0.42
Renal pelvis	Conventional (HU)	74.93	22.61	230.51	60.93	260.74	26.92
	Gold (mg/ml)	2.28	0.51	1.60	0.45	2.16	0.60
	Iodine (mg/ml)	★	★	6.08	1.19	5.80	0.48

Note - data are means ± standard deviation.

★Absence of iodine contrast media.

The presence of iodine could be identified in iodine images, with strong signals in the blood and kidney at T2. These images showed that the signal from iodine in the blood quickly decreased, leaving signal in renal pelvis, matching the expected pharmacokinetics of iodine contrast media. In the kidney and the spleen, the signal decreased quickly from T2 to T3, likely reflecting the rapid clearance from the blood into the urine of this agent. As in the phantom imaging, bone created signal in the iodine and water images.

**Figure 8:**
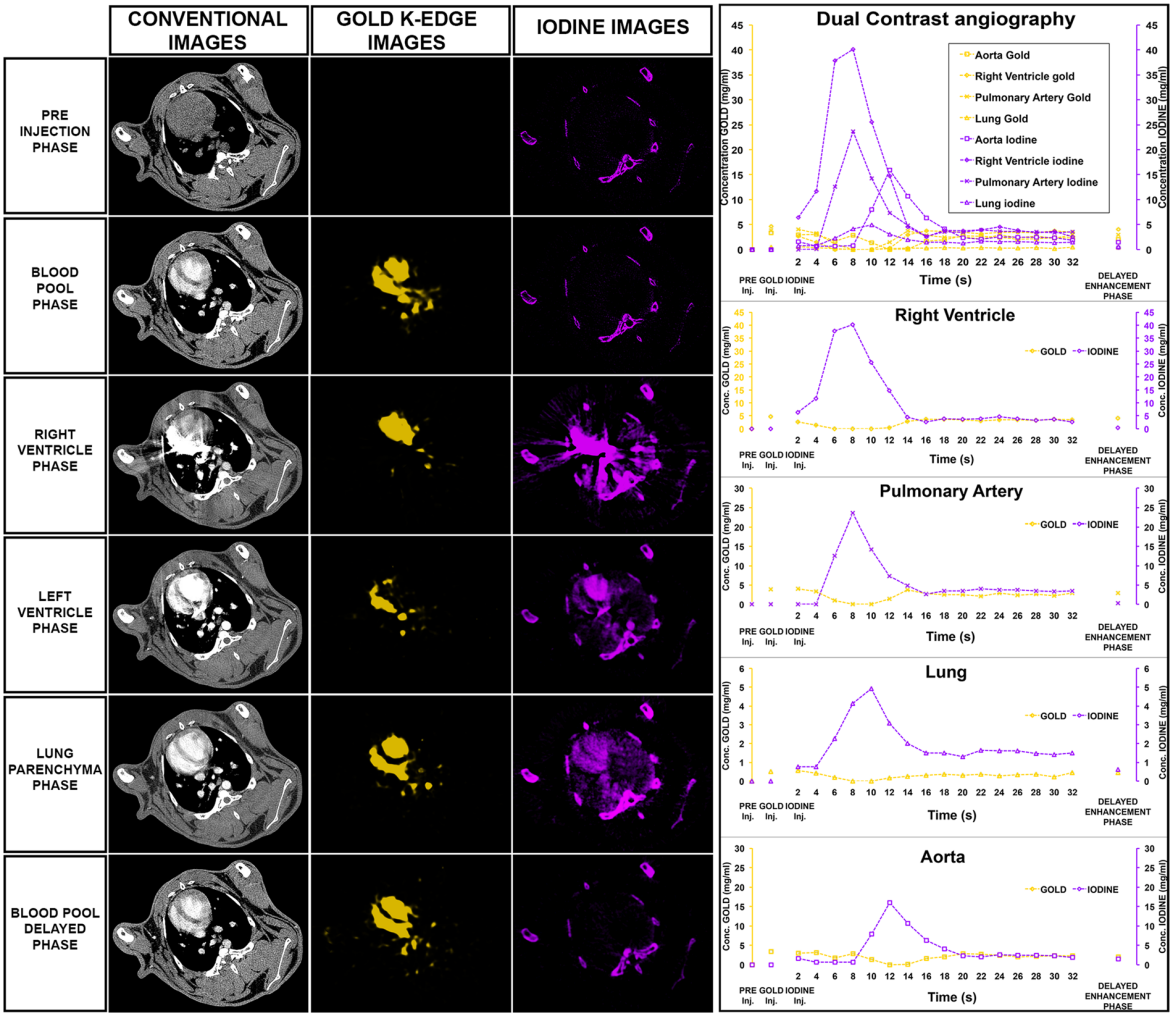
Conventional, gold k-edge and iodine material decomposition images at the level of the heart of a rabbit injected with AuNP and an iodine contrast agent at various timepoints (left). Quantification of the signal in various organs at the different timepoints (right).

The speed of the scanner allowed us to perform multiple repeated scans to capture the pharmacokinetics of the iodine contrast agent immediately post injection. As can be seen in Fig. 8, the bolus of the iodine injection can be tracked from the right ventricle to the pulmonary artery, the lung, and subsequently into the aorta. Such rapidity of scanning has not been demonstrated before to the best of our knowledge and is valuable to be able to assess arterial input function for potential quantification of abnormal tissue perfusion, such in the case of myocardial infarction.

## Discussion

In this study, we demonstrated the ability of a small FOV prototype spectral photon-counting computed tomography system derived from a modified clinical CT system to simultaneously discriminate and quantify a blood pool agent (Au-NP) from an iodinated contrast agent, tissue, and calcium-rich matter on scans of phantoms and in rabbits *in vivo*. In conventional, single energy CT, data is acquired via detectors that measure the energy-integrated signals of x-ray photons, losing energy information of the individual photons and preventing specific discrimination of two contrast agents in one field of view^2, 26, 27^. There are several different configurations for dual-energy CT (dual x-ray tube, fast kVp switching and dual layer detector)^18, 28, 29^, which can provide material specific images. However, these systems produce results of limited spectral performance because the high and low energy datasets are overlapping and the possibilities for material decomposition are limited to an analysis of only two basis images^2, 3^.

In spectral photon-counting CT, information is derived from photon-counting detectors that discriminate photon energies of each detected photon^2, 8^. Therefore, an important advantage used herein of SPCCT over dual-energy CT is that the detected x-rays can be divided into multiple different energy windows to distinguish multiple materials. Additionally one or more boundaries of the energy bins can be freely adjusted to match the K-edge of contrast media such as AuNP or to best match the pathology being imaged^2, 9, 10, 20, 30^. Furthermore, it is generally expected that SPCCT imaging will have lower radiation doses than conventional CT^2, 31^.

Previous studies have focused on the capability of SPCCT to distinguish different materials. These studies were done using systems suited for small animals and were typically conducted postmortem due to the slow scanning speed of these systems^7, 12, 14, 15^. A notable feature of the prototype SPCCT scanner described herein is that it performs axial scans in a clinically relevant time, i.e. one second. There has been a recent report of SPCCT imaging of humans, but multi-material decompositions were not done^21^. The ability to perform absolute quantification of two contrast agents, combined with excellent spatial resolution and fast acquisition times, makes SPCCT a unique imaging modality. SPECT and PET are limited by low spatial resolution^32^ and long acquisition time. Finally, MRI has good spatial resolution but is unable to detect the contrast material itself; it detects the effect on the surrounding protons and this effect is not linearly dependent with the contrast agent concentration^33^.

In this study, the scanned data was processed by material decomposition into the water/iodine/gold bases, therefore calcium-rich matter appears in the iodine and water material images, since all signal must be categorized as one or more of these three materials and the attenuation profiles of water and iodine are closest to that of calcium. K-edge imaging of iodine is not possible with this scanner, since it is a prototype for eventual clinical scanners and therefore low energy (15–30 keV) photons are not used, a distinction from earlier small animal scanners. Therefore, there are too few photons below the K-edge of iodine (33.2 keV) to allow K-edge imaging. Four material basis decomposition into water/iodine/gold/calcium could be done, but has drawbacks of reduced image quality, therefore we performed the afore-mentioned three material decomposition. Nevertheless, knowledge of anatomy and the synthesized conventional CT images can be used to determine whether signal is arising from calcified tissue rather than iodinated contrast media. The specific K-edge imaging of gold did not experience cross-talk from other components in the field of view, accurately discriminating the calcium-rich matter, all other tissue, and also the iodine contrast agent. When mixing two contrast agents, we observed a systematic underestimation of the gold concentration compared with the unmixed contrast agent. This is likely due to imperfections and systematic errors in the current system’s hardware and software, such as the spectral forward model, i.e. uncertainties in the knowledge of the x-ray tube spectrum, as well as in the response function of the detector. Future work will seek to minimize such errors and improve quantification.

We were able to specifically image the biodistribution of an iodine contrast agent, detecting its presence in and quantifying its elimination from the blood vessels. We observed that the agent was rapidly excreted into the renal pelvis, matching the expected pharmacokinetics. We were also able to specifically image the biodistribution of a gold nanoparticle blood pool agent. We found persistently high concentrations of the gold nanoparticles in the blood vessels over the duration of the experiment (41 minutes), allowing both arterial and venous mapping. Previous reports indicate that such nanoparticles will remain in the circulation for several hours^25^, allowing imaging to be done over a wide time window. SPCCT imaging allows the use of blood pool agents for delayed steady-state imaging, and can simultaneously perform first pass arterial imaging using a different contrast agent, such as iodine as we have demonstrated. For instance, it could be interesting to assess liver perfusion and blood mapping simultaneously. Further applications of blood pool contrast agents include detection of bleeding^34^, visualization of tumor vasculature^35^ and quantification of tissue blood volume^36^. Interestingly, we observed a slight decrease in gold concentrations in the blood just after injection of iodine media contrast (T2), with a return to the initial level at T3, which could be due to a bolus effect from the iodine contrast media temporarily displacing the AuNP out of the organs or the reduction of gold signal observed from the second phantom (Fig. 4). We observed some gold signal in the renal pelvis, Since the size of this nanoparticle is above what is considered to be “renally excretable” (5.5 nm)^37^ it seems unlikely that the agent is being filtered and excreted by the kidney, and chemical analysis indicated that gold was not present in the urine. Possibly this signal is due to AuNP in the blood present in this tissue. As a preclinical agent, an important issue for the translation of the gold nanoparticles is toxicity assessments. The formulation used in this study has been shown to be highly biocompatible by several groups^20, 34^, although testing in a non-rodent species would need to be done prior to clinical trials.

This was an early *in vivo* study to probe whether spectral imaging with two contrast media is feasible with a small FOV prototype spectral photon-counting computed tomography system derived from a modified clinical CT system; therefore the sample size we used was small. Moreover, we did not compare the measured concentrations *in vivo* with blood draws, and so we can only rely on the phantom measurements for calibration. Another limitation is that we did not study human subjects. The small FOV of the current scanner is another limitation, however a FOV typical of clinical scanners could be achieved via expansion of the detector array. The principal impediment is that AuNP are not currently clinically approved for use in humans. However, efforts to clinically translate metal-based nanoparticles that will provide spectral CT specific contrast are ongoing^38, 39^.

### Summary

In summary, to the best of our knowledge, we have demonstrated for the first time the potential of the newly developed prototype spectral photon counting CT imaging system for qualitative and quantitative discrimination of two contrast agents *in vivo*, simultaneously over time, indicating the clinical impact of SPCCT in the field of “multicolor” imaging. This finding points to preclinical and clinical applications with various types of contrast agents to probe other biological processes and diseases, and also for multi-phase imaging in a single scan. In addition, it highlights the need to develop SPCCT specific contrast agents, which could expand the field of CT-based molecular imaging.

## Materials and Methods

### Gold nanoparticle preparation and characterization

Polyethylene glycol–coated gold nanoparticles were prepared and characterized as previously described^40, 41^. In brief, AuNP were synthesized in house using the Turkevich method, i.e. by reduction of gold chloride in boiling water via addition of sodium citrate. These nanoparticles were then capped with thiol-PEG-2000 and purified by washing in molecular weight cut-off concentrator tubes with phosphate-buffered saline. The AuNP were concentrated to about 65 mg/ml and sterilized via filtration before use. The nanoparticles were characterized with transmission electron microscopy, dynamic light scattering and inductively coupled plasma-optical emission spectroscopy.

### SPCCT Scanner

The SPCCT scanner (Philips Healthcare, Haifa, Israel) is a prototype spectral photon-counting computed tomography system derived from a modified clinical CT system with a field-of-view (FOV) of 168 mm in-plane, and a z-coverage of 2 mm. It is equipped with energy-sensitive photon-counting detectors made of the direct conversion high band gap semiconductor cadmium zinc telluride. The electronics comprises 5 rate counters with 5 different configurable energy thresholds. For this study the thresholds were set to 30, 51, 78, 83 and 98 keV, chosen to give high differential sensitivity to materials with differing Z’s, and in particular to analysis of gold, which has a K-edge of 80.7 keV. A full description of the SPCCT system can be found elsewhere^42^. Spectral photon-counting CT acquisitions were performed with a conventional X-ray tube at 100 mA tube current and 120 kVp tube voltage with a gantry rotation time of 1 s and 2400 projections per rotation. A generalized schematic of how SPCCT systems operate is shown in Fig. 1A. A photograph of the system is shown in Fig. 1B.

### Material decomposition and quantitative measurements

The material decomposition process was based on a method originally developed for dual-energy CT^43^ that was extended to photon-counting CT^44^, which is independent from the conventional images. The conventional CT images and material decomposition images were derived from SPCCT data using the detected information of the transmitted spectrum in all energy bins. The material decomposition from the multi-bin photon-counting data included a maximum-likelihood based material decomposition of the attenuation into three material images, i.e. water, iodine and gold^45^. The material decomposition process provided water, iodine and gold images in mg/ml, allowing measurements of the concentration of a specific material. The conventional CT equivalent images are provided in Hounsfield units. Images were reconstructed as 0.25 × 0.25 × 0.25 mm voxels using conventional filtered back-projection without further post-processing and a standard Kernel filter.

### Image analysis

For the phantom study, image analysis was performed using MATLAB (MathWorks. Inc.). Samples were automatically detected on conventional images and circular regions of interest were automatically drawn in the middle of each tube. The same ROIs were used on all images generated on a given data set and mean and standard deviation were computed for each ROI. *In vivo* images were analyzed using ImageJ software^46^. The attenuation values in Hounsfield Units (HU) and the concentrations of iodine and gold were recorded from a 2 mm slice, by manually drawing regions of interest (ROIs) of at least 50 pixels in all rabbits for selected organs (i.e. the left ventricle. right kidney. renal pelvis and spleen). The ROI’s were defined on conventional CT images prior to retrieval of the gold and iodine concentrations per organ to avoid operator bias. After obtaining the respective concentrations, no adjustments of the ROIs were made. The ROIs were manually traced in the organs of interest and then were automatically copied on the gold specific K-edge and iodine material decomposition images. Representative ROIs used for image analysis are displayed in Supplementary Figure 3. The data presented is the absolute mean concentration of gold and iodine in mg/ml (mean ± standard deviation) among the five rabbits.

### Phantom imaging

In order to study the material discriminating capabilities of the SPCCT system, we first performed phantom imaging on solutions of polyethylene glycol coated AuNP and an iodinated contrast agent (Iomeron 400 mg/ml. Bracco). A polyoxymethylene cylindrical phantom with a diameter of 13 cm and 12 holes of 1.5 cm in diameter was used. The first phantom contained a range of concentrations of AuNP and iodine contrast media, including 5 different concentrations of gold nanoparticles (2, 4, 6, 8 and 10 mg/ml), 4 concentrations of iodine contrast agent (4, 6, 8 and 10 mg/ml), a phosphate buffered saline (PBS) sample and a calcium phosphate sample (with an attenuation of 1860 HU), all prepared in 1.5 ml polypropylene centrifuge tubes (Dominique Dutscher SAS, Brumath, France) and inserted into the phantom. For the second phantom, eleven 1.5 ml polypropylene centrifuge tubes were prepared, each contained a solution of AuNP mixed with the iodinated agent, in varying proportions and diluted in PBS. The proportions of each solution were adjusted based on data obtained from imaging unmixed contrast agent; the attenuation of each solution was 280 HU at 120 kVp in conventional CT images; the concentration of AuNP and iodine varied between 0–10.4 mg/ml, and 0–8 mg/ml respectively. The concentration ranges for the clinically available iodinated agent was based on current practice, i.e. below the maximum concentrations achievable during the arterial phase (~12–20 mg/ml)^33^.

### Animal imaging

This study was approved by the relevant Institutional Animal Care and Use Committee (Local Ethics Commitee: CeLYne C2EA42, Council Directive No. 2010/63/UE on the protection of animals used for scientific purpose) under the authorization number APAFIS#1732-2015091411181645v3 and performed in accordance with relevant guidelines and regulations. Five adult New Zealand White rabbits (Charles River, Canada, mean weight, 2.8 ± 0.6 kg; 2 females, 3 males; mean age, 6.1 ± 3.0 months) were sedated before imaging using a 20 mg/kg injection of ketamine (10 mg/ml, Merial, Lyon, France) and 0.25 mg/kg injection of medetomidine (1.0 mg/ml, Orion Pharma, Orion Corporation, Espoo, Finland) in order to maintain general anesthesia for 90 minutes. A 22-gauge catheter was placed in the ear vein for contrast agent administration, 3.5 ml/kg (about 12 ml) of AuNP were injected first, followed 25 minutes later by 0.9 ml/kg of Iomeron (3 ml). An initial acquisition was performed pre-injection (time point “T0”), followed by an acquisition at 10 minutes after injection of AuNP (time point “T1”). Subsequent acquisitions were performed 1 (time point “T2”) and 15 minutes (time point “T3”) after injection of iodine media contrast (Fig. 1C). Axial scans were acquired at the level of the heart, liver, spleen and the kidneys, allowing visualization of thoracic blood vessels, cardiac cavities, liver, spleen and renal pelvis. The pharmacokinetics of the iodine contrast agent were imaged by performing scans at the level of the heart every two seconds fifteen times after injection. Image analysis is described above.

### Data Analysis Statistics

For the phantom study, linear regression was used to assess the correlation between the measured and the expected concentrations.

## Electronic supplementary material

Supplementary information
