# Supplementary material for: Multicolor spectral photon-counting computed tomography: in vivo dual contrast imaging with a high count rate scanner
Source: Sci Rep. 2017 Jul 6;7:4784. doi: 10.1038/s41598-017-04659-9 (PMC5500581; doi:10.1038/s41598-017-04659-9)
Supplement: Supplementary file 1 — Supplementary information [file 41598_2017_4659_MOESM1_ESM.pdf]

Supplementary information: Multicolor spectral photon-counting computed tomography: in vivo dual contrast imaging with a high count rate scanner

Authors: David P. Cormode DPhil,[1,**] Salim Si-Mohamed MD,[2,3,**] Daniel Bar-Ness BSc,[2,3] Monica Sigovan PhD,[2,3] Pratap C. Naha PhD,[1] Joelle Balegamire PhD,[4] Franck Lavenne BSc,[5] Philippe Coulon PhD,[6] Ewald Roessl PhD,[7] Matthias Bartels PhD,[7] Michal Rokni PhD,[8] Ira Blevis PhD,[8] Loic Boussel MD PhD,[2,3] Philippe Douek MD PhD[2,3]*

1. Department of Radiology, University of Pennsylvania, Philadelphia, PA, USA
2. Radiology Department, Hospices Civils de Lyon, Lyon, France
3. CREATIS, UMR CNRS 5220, Inserm U1044, University Lyon1 Claude Bernard, Lyon, France
4. LAGEP Laboratory, University Lyon 1 Claude Bernard, Lyon, France
5. CERMEP, Lyon, France
6. CT Clinical Science, Philips, Suresnes, France
7. Philips GmbH Innovative Technologies, Research Laboratories, Hamburg, Germany
8. Global Advanced Technologies, CT, Philips, Haifa, Israel

**** These authors contributed equally to this work**

***Corresponding author**: Philippe Douek

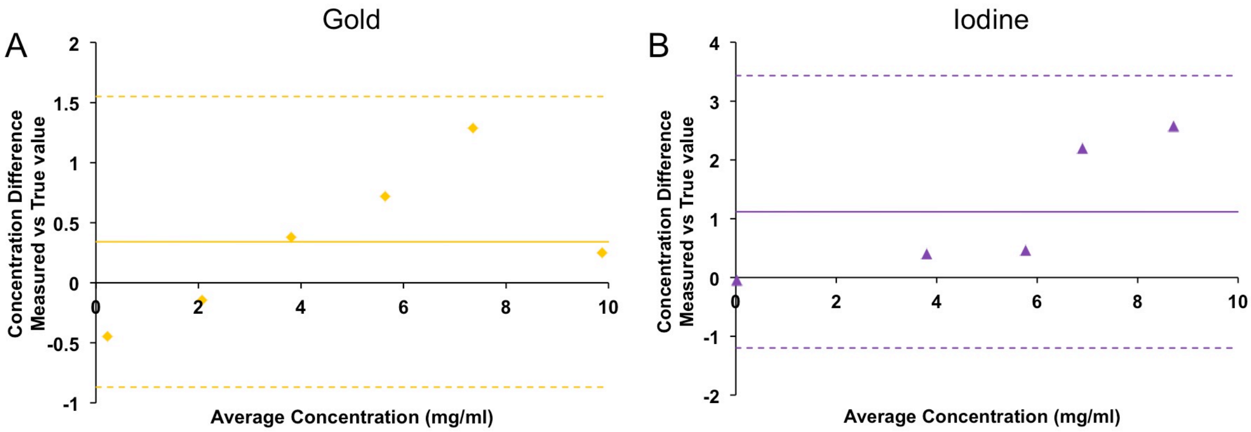

Supporting Figure 1 Bland-Altman plots for the phantom data from Figure 3.

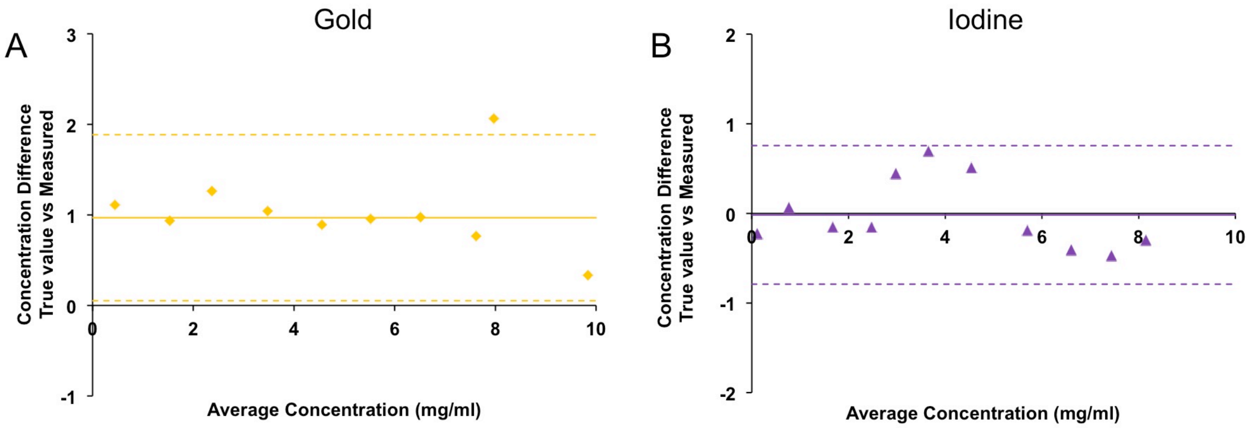

Supporting Figure 2 Bland-Altman plots for the phantom data from Figure 4.

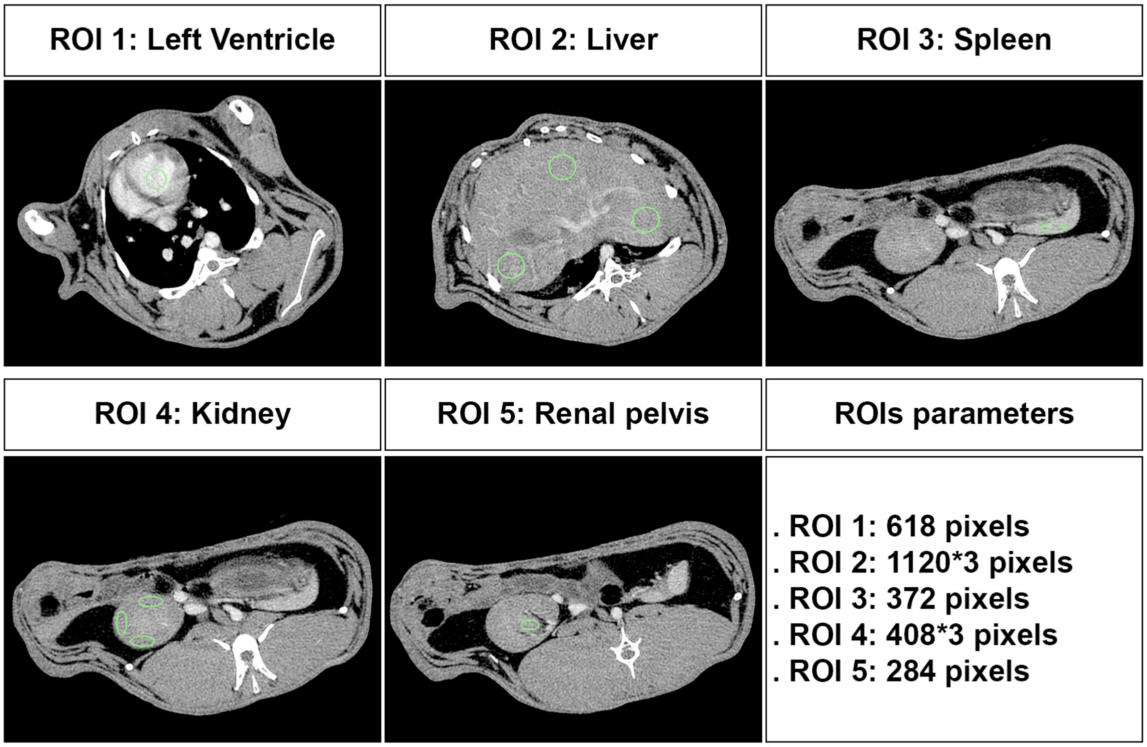

Supporting Figure 3 CT images of a rabbit injected with AuNP and iodine contrast agents with representative ROIs for data analysis depicted.
